# Invivo generated autologous plasmin enzyme assisted vitrectomy, partial circumferential-oral retinotomy, silicone oil injection in patients with chronic retinal detachment without posterior vitreous detachment

**DOI:** 10.1007/s00417-024-06466-1

**Published:** 2024-04-17

**Authors:** Cengiz Aras, Fevzi Senturk, Sevil Karaman Erdur, Mahmut Dogramaci, Mehmet Selim Kocabora, Ali Demircan, Yunus Emre Budak

**Affiliations:** 1 Medicine Faculty, Department of Ophthalmology, Istanbul Medipol University, TEM Göztepe Cıkısı No:1 Bagcilar, 34214 Istanbul, Turkey; 2 Department of Ophthalmology, The Princess Alexandra Hospital NHS Trust, London, UK

**Keywords:** Chronic retinal detachment, Autologous plasmin enzyme, Posterior vitreous detachment, Vitrectomy

## Abstract

**Purpose:**

To report the results of invivo generated autologous plasmin enzyme(IVAP) assisted vitrectomy, partial circumferential-oral retinotomy and silicone oil injection for surgical treatment of patients with chronic retinal detachment without posterior vitreous detachment(PVD).

**Methods:**

Study was performed in retrospective, comparative manner. A total of 16 consecutive eyes with chronic retinal detachment who had intravitreal injection of 50 µgr of t-PA and 0.1 ml of autologous whole blood, 3 days before surgery, underwent lens extraction with phacoemulsification, IVAP assisted vitrectomy, partial circumferential-oral retinotomy, and silicone oil injection(Study Group) were compared to a similar group of 15 eyes who had undergone vitrectomy, with or without lens extraction and silicone oil injection(Control Group) for the treatment of chronic retinal detachment. Primary outcome measures were initial retinal reattachment and number of operations at postoperative 6 months.

**Results:**

Mean age of 16 patients of whom 7 were female, was 39.31 ± 17.76 years in study group and 15 patients of whom 4 were female, was 35.40 ± 11.92 years (*p* = 0.607). Mean follow-up time was 10.68 ± 7.15 months in study group and 29.13 ± 18.83 months in control group (*p* = 0.001). Initial retinal reattachment was achieved in 87.50% (14 out of 16 patients) in the study group, whereas it was 46.66% (7 out of 15 patients) in the control group (*p* = 0.017). The mean number of operations for reattachment in the study group was 1.12 ± 0.34, whereas it was 1.46 ± 0.51 in the control group (*p* = 0.039) at postoperative 6 months While the preoperative LogMAR visual acuity was 1.25 ± 0.64, it was 0.53 ± 0.37 at postoperative 6 months in study group (*p* = 0.001). Conversely, in the control group, the preoperative LogMAR visual acuity was 1.22 ± 0.33, it was 1.20 ± 0.89 at postoperative 6 months (*p* = 0.780). At postoperative 6 months,, epiretinal membrane developed in 2 eyes of the study group, 1 eye in the control group, and phthisis bulbi occurred in 1 eye of control group.

**Conclusion:**

IVAP assisted vitrectomy, partial circumferential-oral retinotomy and silicone oil injection is effective and safe for the surgical treatment of chronic retinal detachment without PVD.

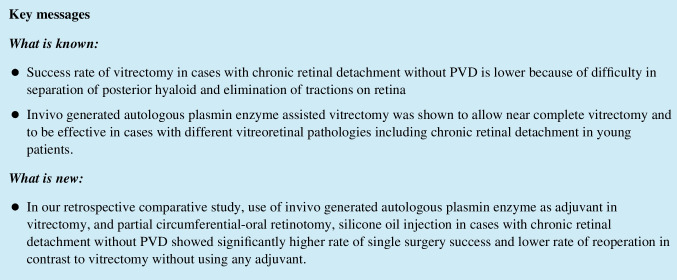

**Supplementary Information:**

The online version contains supplementary material available at 10.1007/s00417-024-06466-1.

## Introduction

Chronic retinal detachment can be described as retinal detachment which is present longer than three months, with clinical findings of pigmented demarcation line, retinal thinning, subretinal bands, intra-retinal cysts and subretinal crystalline opacities [1, 2].

Although, there is no consensus regarding the optimal surgical technique for managing chronic retinal detachment cases, scleral buckling typically emerges as the favored primary approach, exhibiting a notably high success rate [2, 3]. In contrast, vitrectomy is not commonly selected as a primary option due to its comparatively lower success rate and increased probability of necessitating multiple surgical interventions, particularly among younger patients characterized by firm adhesion of the posterior hyaloid to the retina [4].

Autologous plasmin enzyme has been found to facilitate the induction of posterior vitreous detachment (PVD) during vitrectomy procedures in young patients [5–7]. Invivo generated autologous plasmin enzyme (IVAP) assisted vitrectomy was shown to be effective in improving the success rate of vitrectomies performed for various indications, including chronic retinal detachment in young patients [8]. The underlying principle behind the concept of IVAP is based on the coexistence of key components involved in plasmin generation, namely plasminogen and tissue plasminogen activator (t-PA), within the same intraocular environment. Accordingly, prior to surgery, a concurrent injection of t-PA and autologous whole blood into the vitreous cavity takes place three days in advance [8].

In this study, we evaluated the efficacy of IVAP assisted vitrectomy, partial circumferential-oral retinotomy and silicone oil injection in patients with chronic retinal detachment without PVD.

## Materials and methods

The study was performed in a comparative, retrospective, nonrandomized cohort manner. The medical records of cases who underwent vitrectomy for this condition were retrospectively reviewed, encompassing the period from January 2011 to May 2022. The study was conducted at Istanbul University, Cerrahpasa Medical School, and Istanbul Medipol University Hospital, both of which are renowned tertiary referral centers in Turkey. Ethical approval for this study was obtained from the Medical Ethical Committee of Istanbul Medipol University, and the research adhered to the principles outlined in the Declaration of Helsinki (Decision number:555/22.06.2023). Prior to the procedures, written informed consent was obtained from the patients and/or their legal guardians. All surgical interventions were performed by a single experienced surgeon (CA) who has been doing vitreoretinal surgery since 1999.

The study group consisted of 16 consecutive patients who undergone IVAP-assisted vitrectomy, partial circumferential-oral retinotomy, and silicone oil injection for chronic retinal detachment without PVD performed since June 2018 when routine IVAP-assisted vitrectomy was introduced for cases with chronic retinal detachment without PVD. The control group was selected from a comparable cohort, comprising a similar number of patients, who had undergone vitrectomy for chronic retinal detachment prior to June 2018, ensuring pathology matching between the two groups.

Patient characteristics, including age, sex, medical history of ocular and systemic diseases, pre- and postoperative visual acuity, lens status, intraocular pressure, extent of retinal detachment, presence of proliferative changes indicating the chronicity of retinal detachment (such as demarcation line, subretinal band formation, and intra-retinal cysts), history of prior scleral buckling surgery, intraoperative and postoperative complications, reattachment rates and number of reoperations at postoperative 6 months, timing of silicone oil removal, and fellow eye status, were meticulously collected from patient notes. All patients in the study group underwent preoperative color fundus photography and optical coherence tomography (OCT) examinations, which were conducted as part of routine practice at our clinic. Additionally, a subset of patients in the control group also underwent these preoperative assessments, following the same standardized protocols. Hypotony, defined as a persistently low intraocular pressure (IOP) of less than 5 mmHg, was also assessed. Patients with a follow-up duration of less than 6 months and those with a history of ocular trauma, tractional retinal detachment due to proliferative diabetic retinopathy, macula-on detachments were excluded from the study.

Patients in the study group were prepared in a standard clinical setting, following the established protocols for intravitreal injections. This involved a three-minute application of 5% povidone iodine to ensure proper disinfection, followed by draping and the use of a lid speculum to maintain eyelid separation. Subsequently, a small volume (0.1–0.2 ml) of aqueous humor was aspirated from the anterior chamber via paracentesis. Immediately thereafter, 0.1 ml of autologous whole blood, obtained from the brachial vein, was injected into the vitreous cavity at the superior quadrant, approximately 3 mm behind the limbus, using a 27 G needle under topical anesthesia. Additionally, an intravitreal injection of 50 µg of tissue plasminogen activator (t-PA) (Actylise, Boehringer Ingelheim, Ingelheim am Rhein, Germany) in a volume of 0.05 ml was administered using a 30 G needle. Topical antibiotics were prescribed for a three-day period following the injection to minimize the risk of infection.

## Surgical technique

In the study group, the following surgical procedures were performed: lens extraction with phacoemulsification and intraocular lens implantation, 23-G three-port pars plana vitrectomy, posterior hyaloid separation by active aspiration with a vitreous cutter at the nasal side of optic nerve head(see video, Supplemental Digital Content 1 which demonstrates IVAP assisted separation of posterior hyaloid), vitreous base shaving with indentation under Chandelier light illumination, partial circumferential retinotomy spanning 90 to 200 degrees with a vitreous cutter at the ora serrata of the detached quadrant, elevation of the posterior retina to lyse all adhesions, and removal of subretinal membranes using a serrated forceps (see video,Supplemental Digital Content 2 which demonstrates removal of subretinal membranes) Additionally, 360 degrees of laser photocoagulation were performed under the cover of liquid perfluorocarbon. This was followed by a fluid-air exchange and injection of 1000-cSt silicone oil in all patients in the study group. If the presence of an epiretinal membrane was determined by optical coherence tomography (OCT) preoperatively, the surgical procedure of epiretinal membrane and macular inner limiting membrane peeling after staining with a dual blue dye (Twın ALCHIMIA, Italy) was added.

In the control group, both 20-G and 23-G pars plana vitrectomy procedures were performed following the same surgical approach, with the exception of adjuvant use and circumferential-oral retinotomy. Lens extraction with phacoemulsification and intraocular lens implantation, silicone oil tamponade, and 360-degree laser photocoagulation were also carried out in the eyes of the control group.

The primary outcomes of this study were initial anatomic success which was defined as reattachment after one surgery and number of the operations needed for achievement of reattachment at postoperative 6 months. Silicone oil removal surgery was not considered as second surgery. Secondary outcome measures included the change in best corrected visual acuity at the 6-month postoperative follow-up, as well as the assessment of intraoperative and postoperative complications at 6 months.

For statistical analysis, the data on visual acuity taken with the Snellen chart were converted to logarithm of minimum angle of resolution (LogMAR) equivalents. For statistical analysis, the data on visual acuity taken with Snellen Chart was converted to the logarithm of minimum angle of resolution (LogMAR) equivalents. The results from both groups were analyzed and compared using Pearson Chi-Square test, Student t test, Mann Whitney U-test and Wilcoxon Signed test (Statistical Package for Social sciences, version 26). The significance level was set to *p* < 0.05.

## Results

In this study, we compared a study group consisting of 16 consecutive eyes that underwent IVAP-assisted vitrectomy, partial circumferential oral retinotomy, subretinal membrane removal (when applicable), and silicone oil injections to a control group of 15 eyes with chronic retinal detachment who had undergone vitrectomy with or without lens extraction and silicone oil injection. The baseline characteristics of the two groups are presented in Table 1.

**Table 1:** Baseline characteristics of the patient population

	Study Group	Control Group	P-value
No. of eyes	16	15	-
Sex: F/M [no.]	7/9	4/11	0.328^a^
Age (years) (Range)	39.31 ± 17.76 (14 to 73)	35.40 ± 11.92 (17 to 52)	0.607^c^
Laterality: Right/Left	6/10	6/9	0.541^a^
Prior Buckling Surgery	4/16	8/15	0.106^a^
Lens status Clear Lens Cataract Pseudophakia	14/16 - 2/16	13/15 - 2/15	0.689^a^
Follow-up time (months) (Range)	10.68 ± 7.15 (6 to 26)	29.13 ± 18.83 (6 to 72)	0.001^c^
Pre-op VA (LogMAR)	1.25 ± 0.64 (0.52 to 3.10)	1.22 ± 0.33 (0.40 to 1.80)	0.819^b^
Macular Involvement	15/16	15/15	1.00^a^
Involved Quadrant			
Superior İnferior Four Quadrants	2 10 4	3 9 3	0.842^a^
Preoperative Findings of Chronic RD			
Demarcation Line Subretinal Band Formation Intraretinal Cyst Formation	11/16 14/16 2/16	12/15 12/15 3/15	0.482^a^ 0.577^a^ 0.577^a^

F/M: Female/Male, RD: Retinal Detachment

^a^Chi-square test

^b^Mann-Whitney U- test

^c^Student-t test

**Fig. 1:**
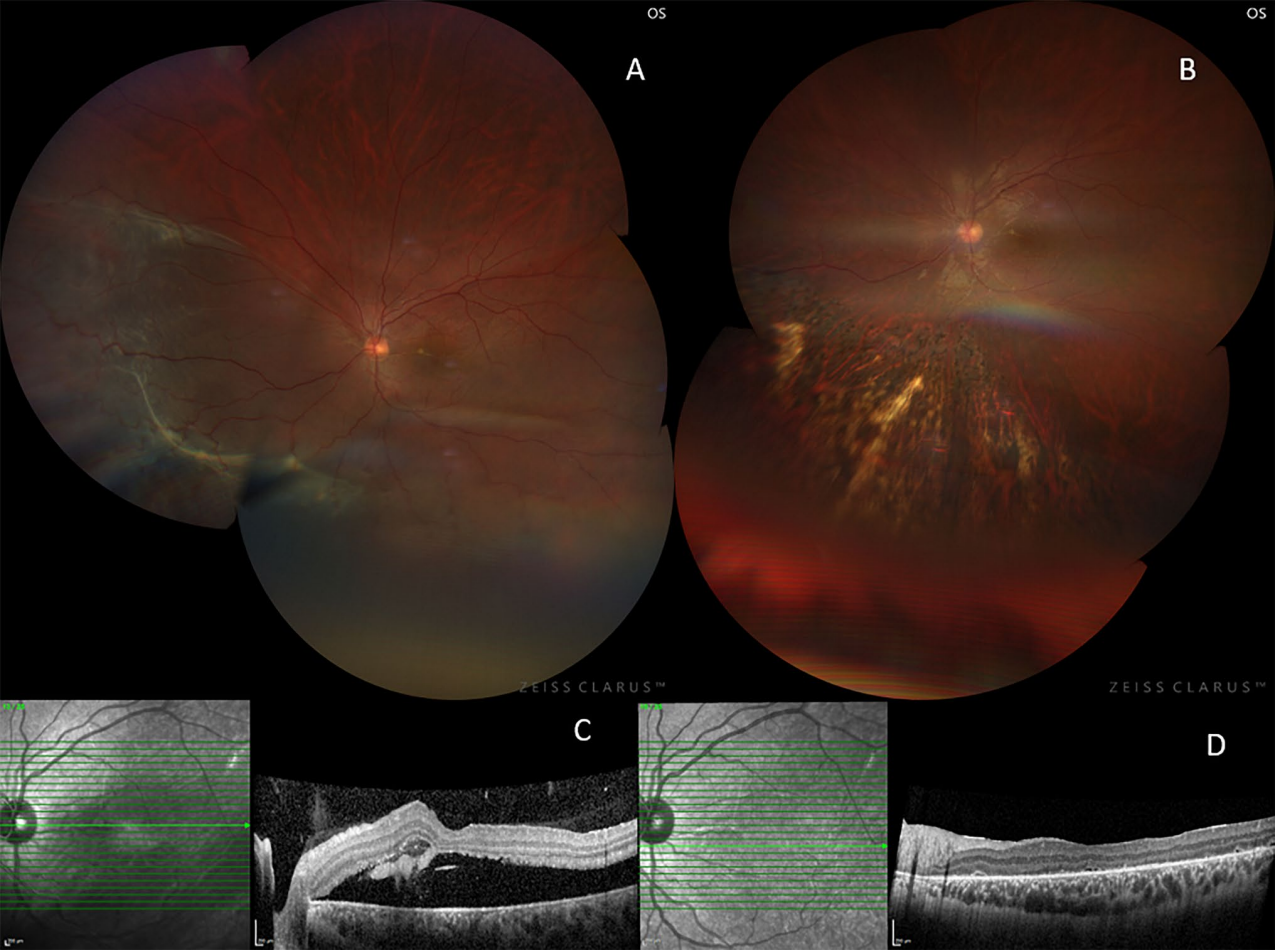
Pre (**A**) and postoperative (**B**, **D**) colour pictures and OCTs of a patient with chronic retinal detachment from study group. Note that posterior vitreous was not detached before surgery (**C**)

**Fig. 2:**
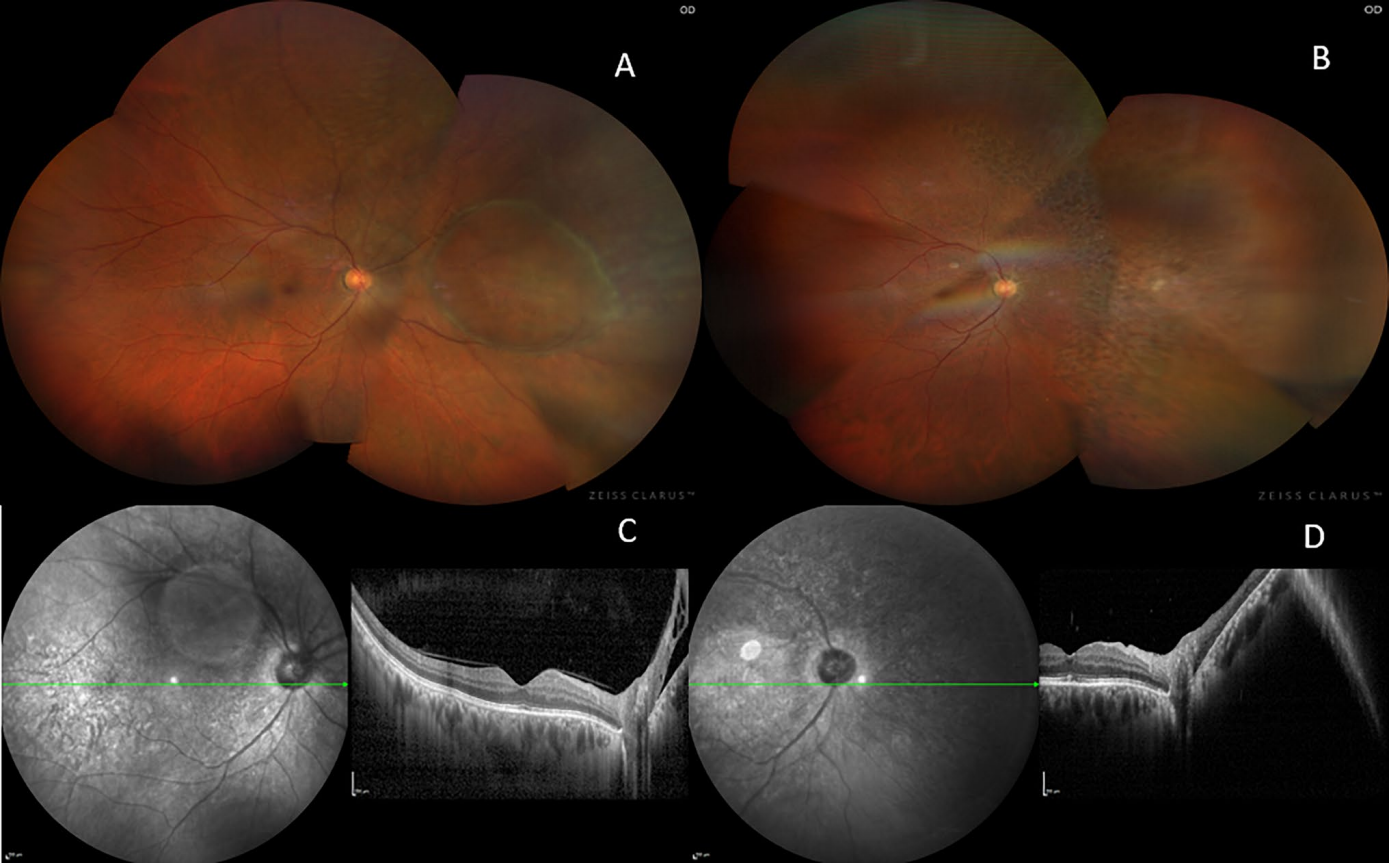
Pre (**A**) and postoperative (**B**, **D**) colour pictures and OCTs of a patient with chronic retinal detachment from study group. Note the existence of intraretinal cyst at detached retina at nasal side of optic nerve head and unseparated posterior hyaloid (**C**)

No statistically significant differences were found in the preoperative baseline characteristics between the two groups. The mean age of patients in the study group was 39.31 ± 17.76 years (range: 14 to 73), while in the control group, it was 35.40 ± 11.92 years (range: 17 to 52) (*p* = 0.607). In the study group, 56% (*n* = 9) of patients were male, compared to 73% (*n* = 11) in the control group (*p* = 0.328). Both groups had two pseudophakic eyes, while the remaining eyes had no cataracts (*p* = 0.689). In the study group, all phakic eyes underwent lens extraction with phacoemulsification and intraocular lens implantation, while 11 out 15 eyes in the control group underwent the same procedure. All the eyes in study group had attached posterior hyaloid which was proven with OCT (Figs. 1 and 2). Prior failed buckling surgery was observed in 25% (*n* = 4) of eyes in the study group and 53% (*n* = 8) of eyes in the control group (*p* = 0.106). Silicone oil was used as a tamponading agent in all eyes of both groups. The mean follow-up time was 10.68 ± 7.15 months (range: 6 to 26) in the study group and 29.13 ± 18.83 months (range: 6 to 72) in the control group (*p* = 0.001).

The mean number of operations in the study group was 1.12 ± 0.34, whereas it was 1.46 ± 0.51 in the control group (*p* = 0.039) at postoperative 6 months. Initial anatomic reattachment was achieved in 87.50% of eyes (14 out of 16 eyes) in the study group, whereas it was 46.66% (7 out of 15 eyes) in the control group (*p* = 0.017) (Table 2). Final anatomic success was achieved in 93.75% (15 eyes) in the study group and 73.33% (11 eyes) in the control group (*p* = 0.172).

**Table 2:** The results of primary outcome measures at postoperative 6 months

Primary Outcome Measures	Study Group	Control Group	*P*-value
Single Surgery Success	14/16 (87.50%)	7/15 (46.66%)	0.017^a^
Mean Number of Operation	1.12 ± 0.34 (1 to 2)	1.46 ± 0.51 (1 to 2)	0.039^b^

^a^Chi-square test

^b^Mann-Whitney U- test

In the study group, the preoperative LogMAR visual acuity was 1.25 ± 0.64 (range: 0.52 to 3.10), which improved to 0.53 ± 0.37 (range: 0.10 to 1.30) at the 6-month postoperative follow-up (*p* = 0.001). Conversely, in the control group, the preoperative LogMAR visual acuity was 1.22 ± 0.33 (range: 0.40 to 1.80), and it was 1.20 ± 0.89 (range: 0.15 to 3.10) at the 6-month postoperative follow-up (*p* = 0.780) (Table 3). The mean change in best corrected visual acuity was -0.72 in the IVAP-assisted vitrectomy group, while it was -0.02 in the control group. This difference was statistically significant (*p* = 0.008).

**Table 3:** Pre- and postoperative 6 months visual acuity

Visual acuity [logMAR]	Preoperative mean(range)	Post-op 6 months mean(range)	*P*-value
Study Group	1.25 ± 0.64 (0.52 to 3.10)	0.53 ± 0.37 (0.10 to 1.30)	0.001^a^
Control Group	1.22 ± 0.33 (0.40 to 1.80)	1.20 ± 0.89 (0.15 to 3.10)	0.780^a^

^a^Wilcoxon Signed test

Silicone oil was removed from all eyes in the absence of hypotony, and its removal was not considered an additional surgical procedure. The mean duration of silicone oil tamponade was 8.26 ± 2.43 weeks in the study group and 11.36 ± 4.52 weeks in the control group (*p* = 0.07). At the last follow-up examination, one eye in the study group and two eyes in the control group still had silicone oil tamponade due to persistent hypotony.

Intraoperatively, we observed the presence of some amount of blood under the retina, which passed through the breaks in almost all cases in the study group. The removal of blood did not pose difficulties for the surgeon, as easy access to the subretinal space was achieved through the pre-planned partial circumferential-oral retinotomy during surgical intervention. Persistent subretinal fluid developed in four eyes in the study group and three eyes in the control group, but it regressed within six months in all cases. Two eyes in the study group and 7 eyes in the control group experienced recurrent retinal detachment due to proliferative vitreoretinopathy, requiring redo vitrectomy at postoperative 6 months. Additionally, epiretinal membrane formation was developed in 2 eyes of the study group, 1 eye in the control group, and phthisis bulbi occurred in 1 eye of control group (Table 4).

**Table 4:** Postoperative complications in both groups at 6 months

Complications	Group 1	Group 2	P-value
Epiretinal membrane	2/16(12.50%)	1/15(6.66%)	0.022^**a**^
Persistent Subretinal Fluid	4/16(25%)	3/15(20%)	
Persistent Hypotony	1/16(6.25%)	1/15(6.66%)	
Retinal Detachment	2/16(12.50%)	7/15(46.66%)	
Phthisis bulbi	0/16(0%)	1/15(6.66%)	

^**a**^Fischer Exact test

## Discussion

The clinical course and outcomes of acute rhegmatogenous retinal detachment are well studied. However, the outcomes of chronic retinal detachment are not clear enough mainly due to its low incidence. Therefore, choice of the optimal surgical technique for the treatment of chronic retinal detachment remains a topic of debate and lacks consensus. Given age factor, phakic status, and the presence of an undetached posterior vitreous, scleral buckling is commonly regarded as the surgical approach of choice in chronic retinal detachment without PVD [2, 3]. Conversely, primary vitrectomy is typically reserved for cases presenting with posteriorly located retinal breaks, a contracted retina due to intraretinal fibrosis, associated with gigantic intraretinal cyst formation, or following unsuccessful prior scleral buckling surgery.

Vitrectomy procedures in chronic retinal detachment without PVD pose significant challenges due to the difficulties in achieving successful surgical induction of PVD in younger patients, complete removal of the vitreous, and the relaxation of tractions related to subretinal membranes and intraretinal contraction. In the context of addressing these challenges, the utilization of in vivo generated autologous plasmin enzyme as adjuvant during vitrectomy has shown promising results in increasing the success rate of the procedure for various indications, including chronic retinal detachment in young patients [8].

In our study, we used preoperative adjuvant that involved simultaneous intravitreal injection of 50 µg of tissue plasminogen activator (t-PA), along with 0.1 ml autologous whole blood as a source of plasminogen which is main substrate for plasmin generation, three days prior to the surgery. This approach facilitated the separation of the posterior hyaloid from the retina with the help of the generated autologous plasmin enzyme in vitreous cavity, enabling us to perform more comprehensive vitrectomy along with concurrent lens extraction in our cases. The blood in vitreous cavity is generally considered as stimulating factor for proliferative vitreoretinopathy. But, we injected blood 3 days before surgery which is too short time to cause proliferation. Subsequently, we conducted primary circumferential-oral retinotomy in the detached quadrant and, when necessary, retinectomy in the presence of retinal fibrosis. These interventions aimed to address the antero-posterior traction caused by intraretinal contraction and to remove subretinal membranes. In term of outcomes, comparing this approach to the control group, where no prior adjuvants were used and no retinotomy was planned, the new approach consistently achieved a higher rate of initial retinal reattachment.

In the surgical management of retinal detachment complicated with proliferative vitreoretinopathy (PVR), various studies have demonstrated a high rate of reattachment when employing the technique of circumferential retinotomy-retinectomy [9, 10]. The concept of peripheral, clean, straight, and large retinotomies, originally described by Machemer [11], aims to address both intraretinal anteroposterior contraction and subretinal membranes. In our study, following near complete vitrectomy, we performed partial circumferential-oral retinotomy in order to alleviate antero-posterior traction and remove subretinal membranes in the study group. We did not combine vitrectomy with scleral buckling; however, in eyes that underwent vitrectomy following prior failed buckling surgery, the buckles were not removed. Our rationale for utilizing partial circumferential retinotomy was to prevent potential peripheral contraction of the retina, assuming that near complete vitrectomy had been achieved.

The improvement in outcome seen in patients who underwent IVAP assisted vitrectomy and partial circumferential oral retinotomy is primarily due to a more through release of the tractions on the retina which in turn reduces the risk of recurrence of detachment. It has previously been suggested that inadequate removal of the vitreous during vitrectomy could lead to the recurrence of retinal detachment through the proliferation of residual vitreous which could impose further traction on the retina leading to retinal breaks or reopening of the existing ones [12, 13]. In our view, the synergetic action of IVAP assisted vitrectomy and lens extraction ensures a near complete removal of vitreous humor.

It was shown that silicone oil tamponade has higher anatomic success rate and lower hypotony rates when compared to other tamponades in eyes that underwent retinotomy [14, 15]. Therefore, we used silicone oil tamponade in all cases of both groups. In our series, those who underwent IVAP assisted vitrectomy and partial circumferential-oral retinotomy showed a significant reduction in the number of total operations, a significant improvement in vision recovery, and significant increase in single surgery success rates.

Recently, chronic macula-off retinal detachments are delineated into with PVD and without PVD [16]. Those without PVD have significantly lower rates of initial reattachment and high rates of PVR. All the eyes in our study group had preoperatively attached posterior hyaloid which were based on OCT imaging. That is why we used IVAP for facilitating intraoperative separation of posterior hyaloid in all the patients of the study group.

Limitations of our study were its retrospective, nonrandomized manner, relatively small sample size due to low incidence of chronic retinal detachment. Additionally, differences in follow up duration, potential influence on accessibility of the peripheral retina and on visual acuity outcomes of postoperatively phakic eyes in the control group and lack of standardization of some surgical steps between groups were other limitations of our study.

In conclusion, this study provides early evidence that IVAP assisted vitrectomy, partial circumferential-oral retinotomy may improve single surgery success rate in chronic retinal detachment without PVD, but larger prospective, randomized studies with longer follow-up are needed to better evaluate this potential technique for the treatment of chronic retinal detachment without PVD.

## Supplementary Information

Below is the link to the electronic supplementary material.

Supplementary file1 (MP4 23966 KB)

Supplementary file2 (MP4 12532 KB)
